# Prognostic value of glycolipid metabolism index on complications and mechanical ventilation in intensive care unit patients with intracerebral hemorrhage: a retrospective cohort study using the MIMIC-IV database

**DOI:** 10.3389/fneur.2025.1516627

**Published:** 2025-02-25

**Authors:** Yile Zeng, Long Lin, Jianlong Chen, Shengyu Cai, Jinqing Lai, Weipeng Hu, Yiqi Liu

**Affiliations:** ^1^Department of Neurosurgery, The Second Affiliated Hospital of Fujian Medical University, Quanzhou, Fujian, China; ^2^Department of Neurosurgery, Fuzong Clinical Medical College, Fujian Medical University, Fuzhou, Fujian, China

**Keywords:** triglyceride-glucose index, atherogenic index of plasma, intracerebral hemorrhage, MIMIC-IV database, complication, mechanical ventilation

## Abstract

**Objective:**

This study aimed to evaluate the predictive capability of glycolipid metabolism index (triglyceride-glucose index, TyG; atherogenic index of plasma, AIP; triglyceride to high-density lipoprotein cholesterol ratio, TG/HDL-C; and non-HDL-C to HDL-C ratio, NHHR) for complications and ventilator use in patients with intracerebral hemorrhage (ICH) admitted to the intensive care unit (ICU).

**Methods:**

Patients with ICH requiring ICU admission were selected from the Medical Information Mart for Intensive Care IV (MIMIC-IV) database. Outcomes assessed included incidence of complications and use of ventilator support. Multivariate logistic regression, receiver operating characteristic (ROC) analysis, and restricted cubic spline were employed to investigate the relationship between glycolipid metabolism index and clinical outcomes in ICH patients.

**Results:**

A total of 733 patients were included. Multivariate logistic regression analysis revealed that elevated TyG, AIP, and TG/HDL-C levels were associated with increased incidence of complications and prolonged ventilator use. ROC curve analysis demonstrated that TyG (AUC 0.646) exhibited the strongest predictive ability for multiple complications in ICH patients. Further multiple regression analysis identified TG/HDL-C as an independent predictor of deep vein thrombosis, while TyG, AIP, and TG/HDL-C independently predicted pulmonary embolism, and TyG, AIP, NHHR, and TG/HDL-C independently predicted acute kidney injury. Moreover, ventilator use further heightened the risk of multiple complications in ICU patients with elevated glycolipid metabolism index.

**Conclusion:**

Glycolipid metabolism index represent promising and readily accessible biomarkers for predicting multiple complications and ventilator use in ICU patients with ICH.

## Introduction

Intracerebral hemorrhage (ICH) stands as a major global contributor to disease burden, characterized by its high mortality and severe neurological impairments, posing significant challenges (1). Although advances in critical care management have resulted in a declining mortality rate from ICH, complications following ICH remain a focus of concern. Current research predominantly focuses on central nervous system complications post-ICH, such as cerebral edema, elevated intracranial pressure, and secondary ischemic injury (2–4). Peripheral complications, however, such as infections (e.g., pneumonia, urinary tract infections, sepsis), thrombotic events (e.g., deep vein thrombosis, pulmonary embolism), and organ dysfunction (e.g., gastrointestinal ulcers, bleeding and acute kidney injury), are frequently overlooked (5, 6). These complications not only complicate ventilator management for critically ill patients but also potentially prolong hospital stays and increase treatment costs (5). The use of a ventilator may increase the risk of medical complications in ICH patients (6), thereby perpetuating a vicious cycle. Hence, effective prediction and management of these complications are paramount, aiming to reduce hospital-acquired complications and thereby improve clinical outcomes and quality of life for patients.

Dysregulation of glucose and lipid metabolism has been implicated in increasing infection risk (7), promoting thrombosis (8), and impacting various organs including the kidneys, heart, and nervous system to varying degrees (9). Recent studies have identified several metabolic index such as triglyceride-glucose index (TyG) (10), atherogenic index of plasma (AIP) (11), non-high density lipoprotein cholesterol to high density lipoprotein cholesterol ratio (NHHR) (12), and triglyceride to high density lipoprotein cholesterol ratio (TG/HDL-C) (13) as potential predictors of adverse outcomes in critically ill patients. However, their specific predictive value in forecasting complications and mechanical ventilation (MV) duration in patients with intracerebral hemorrhage awaits systematic evaluation. In this retrospective cohort study utilizing the Medical Information Mart for Intensive Care IV (MIMIC-IV) database, we aim to assess the prognostic significance of these metabolic index in predicting complications and MV use among patients with intracerebral hemorrhage. By elucidating the role of glucose and lipid metabolism in the prognosis of intracerebral hemorrhage, we hope to contribute to the development of targeted therapeutic strategies, thereby enhancing clinical outcomes and alleviating the burden imposed by this devastating neurological disorder.

## Methods

### Study population

**Figure 1:**
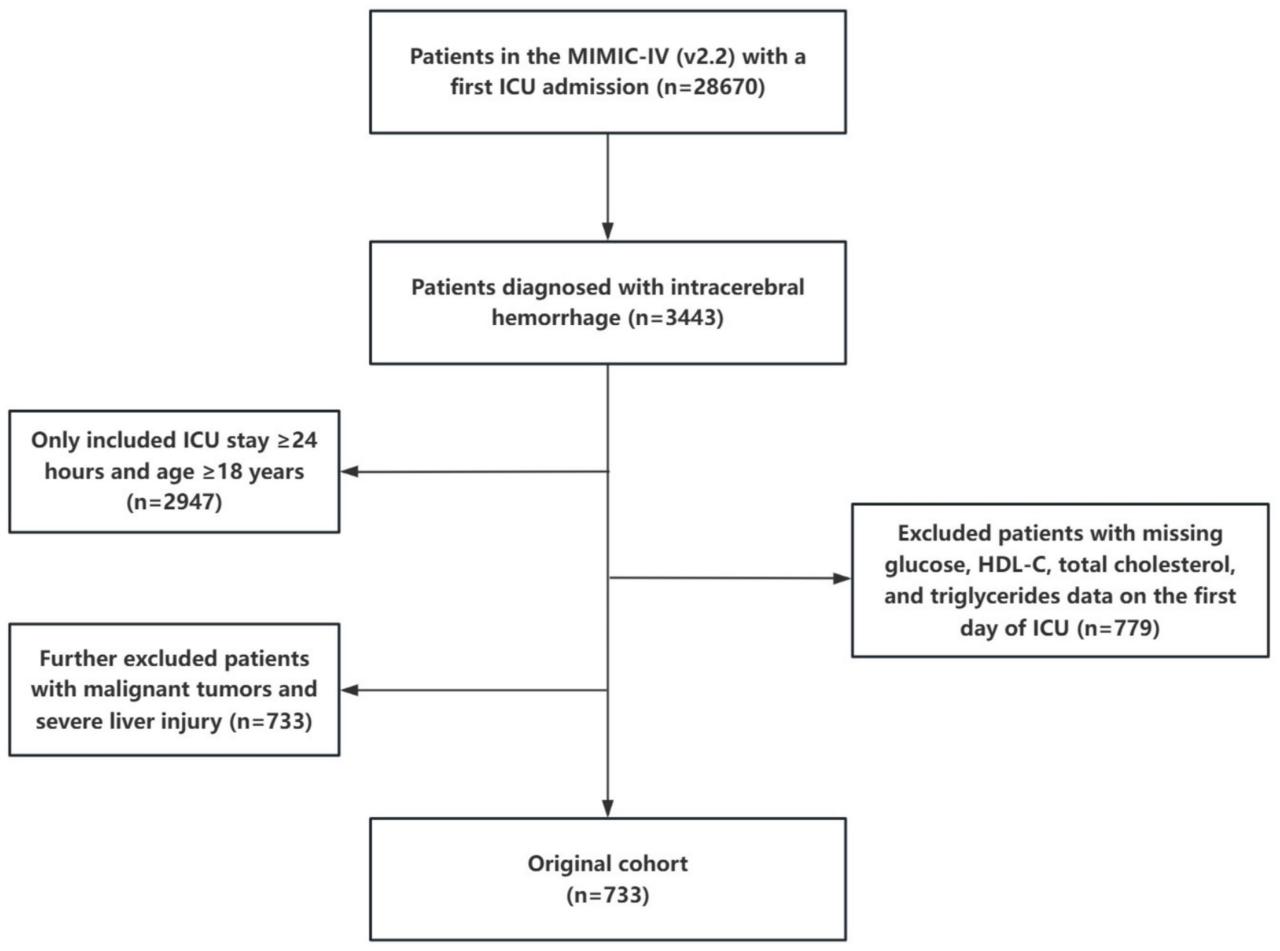
Flow of included patients through the trial.

We conducted a retrospective study using data from the MIMIC-IV database (version 2.2), a large ICU database jointly developed by the MIT Laboratory for Computational Physiology, Beth Israel Deaconess Medical Center and Philips Healthcare. The study included patients diagnosed with ICH based on International Classification of Diseases codes 9th and 10th revisions. As this was a retrospective cohort study, we utilized all available data that met the criteria, aiming for a sufficient sample size to provide statistical power for analyzing the relationships between glycolipid metabolism indices and complications. Inclusion criteria were: (1) patients aged ≥18 years admitted to the ICU for the first time; (2) ICU stay exceeding 24 h; and (3) absence of severe liver or kidney disease and malignancies. Patients with insufficient data, particularly regarding glucose, HDL-C, total cholesterol, and TG, were excluded from the final cohort. A total of 733 patients were included in this study (Figure 1).

### Data collection

We utilized PostgreSQL (version 16.3) and Navicat Premium (version 17) software for data extraction from the MIMIC-IV database, encompassing demographic characteristics, vital signs, laboratory values, complications, and MV status. Specifically, (1) demographic features included age, gender, weight, and race; (2) vital signs encompassed respiratory rate, heart rate, systolic blood pressure, diastolic blood pressure, mean arterial pressure, and peripheral capillary oxygen saturation (SpO2); (3) laboratory indicators comprised white blood cells, red blood cells, platelets, hemoglobin, serum sodium, potassium, calcium, creatinine, blood urea nitrogen, international normalized ratio, partial thromboplastin time, prothrombin time, glucose, HDL-C, total cholesterol, and TG; (4) complications included epilepsy, deep vein thrombosis, pulmonary embolism, gastrointestinal ulceration and bleeding, urinary tract infection, sepsis, and acute kidney injury; and (5) MV status indicated whether MV was used and its duration. All vital signs and laboratory values were derived from the first available data within 24 h of ICU admission.

### Definition

In addition, the following formula was applied to calculate various parameters. TyG = LN [TG × glucose]/2. AIP = Lg[TG/HDL-C]. NHHR = (total cholesterol-HDL-C)/HDL-C. TG/HDL-C = triglyceride to high density lipoprotein cholesterol ratio. Multi-complications: ≥2 of the aforementioned complications. The study participants were stratified according to the median TyG level, median AIP level, median NHHR level, median TG/HDL level in this study and whether to use a ventilator, thus creating subgroups as follow: (1) Low TyG + no ventilator; (2) High TyG + no Ventilator; (3) Low TyG + Ventilator; (4) High TyG + Ventilator; (5) Low AIP + no ventilator; (6) High AIP + no Ventilator; (7) Low AIP + Ventilator; (8) High AIP + Ventilator; (9) Low NHHR + no ventilator; (10) High NHHR + no Ventilator; (11) Low NHHR + Ventilator; (12) High NHHR + Ventilator; (13) Low TG/HDL + no ventilator; (14) High TG/HDL + no Ventilator; (15) Low TG/HDL + Ventilator; (16) High TG/HDL + Ventilator.

### Statistical analysis

All data were presented as mean ± standard deviation or median (interquartile range) for continuous variables and counts (percentage) for categorical variables, respectively. Continuous variables were compared by student’s *t*-test (normally distributed) or Mann–Whitney *U*-test (non-normally distributed). Categorical variables were analyzed using chi-square test or Fisher’s exact test.

Restricted cubic spline (RCS) curve was used to evaluate the correlation between continuous TyG, AIP, NHHR, TG/HDL and multi-complications, respectively. Area under the curve (AUC) was applied to represent the predictive power of TyG, AIP, NHHR, and TG/HDL-C. The study employed linear regression to determine the predictive ability of the four metabolic indices on the number of complications and the duration of the ventilators, adjusting for potential confounders. Both univariate logistic regression and multivariate logistic regression analysis were used to evaluate the association between the four metabolic index and the occurrence of multi-complications and the use of ventilators. In addition, through further multivariate logistics regression, we analyzed the relationship between the four metabolic index and various complications in detail. Finally, we employed multivariate logistic regression again to calculate adjusted odd ratios (ORs) with 95% confidence intervals (CIs) of the four metabolic index and ventilator usage as adjuncts, for the occurrence of multi-complications in the entire cohort. Both multivariate linear regression and multivariate logistics regression analysis were adjusted for the following confounders: age, sex, mean blood pressure, SpO2, congestive heart failure, race, and diabetes.

Statistical analyses were conducted using the R statistical language (version 4.2.1; R Foundation, Vienna, Austria). *p* < 0.05 (two-tailed) was considered statistically significant.

### Ethics statement

The study followed the regulations outlined in the Helsinki Declaration. The MIMIC-IV database de-identifies all patient information and assigns a random code to patient identification. Due to the retrospective nature of the study and anonymity of data, the Ethics Committee of the Second Affiliated Hospital of Fujian Medical University waived the need of obtaining informed consent.

## Results

### Baseline characteristics

The study enrolled a total of 733 ICU-confirmed ICH patients, categorized based on MV use. Baseline characteristics of participants are detailed in Supplementary Table S1. The mean age was 70.3 years, with 396 (54.02%) being male. Overall, 495 patients (67.53%) required MV during ICU stay. MV patients exhibited prolonged ICU hospitalization (*p* < 0.001) and showed higher levels of white blood cells (*p* < 0.001), creatinine (*p* = 0.029), blood urea nitrogen (*p* = 0.020), glucose (*p* < 0.001), triglycerides (*p* = 0.006), lower calcium ion levels (*p* < 0.001), total cholesterol (*p* = 0.016), and HDL-C (*p* = 0.026). In the MV group, TyG (*p* < 0.001), AIP (*p* = 0.002), and TG/HDL-C (*p* = 0.003) were significantly elevated, suggesting a propensity for dysglycemia and dyslipidemia in MV patients. This association may be linked to increased susceptibility to obesity and infections (14, 15). Importantly, MV use was associated with significantly higher risks of multiple complications (*p* = 0.002), including epilepsy (*p* = 0.036), pulmonary embolism (*p* = 0.046), and acute kidney injury (*p* < 0.001). Patients with multiple complications also exhibited higher TyG (*p* = 0.006) and AIP (*p* = 0.024) levels, and had higher likelihood of MV use (*p* = 0.002) and longer MV duration (*p* < 0.001) (Supplementary Table S2). These findings underscore the close association between MV and complications in ICH, potentially linked to underlying disturbances in glycolipid metabolism.

### Relationship between glycolipid metabolism index and complications after ICH

**Figure 2:**
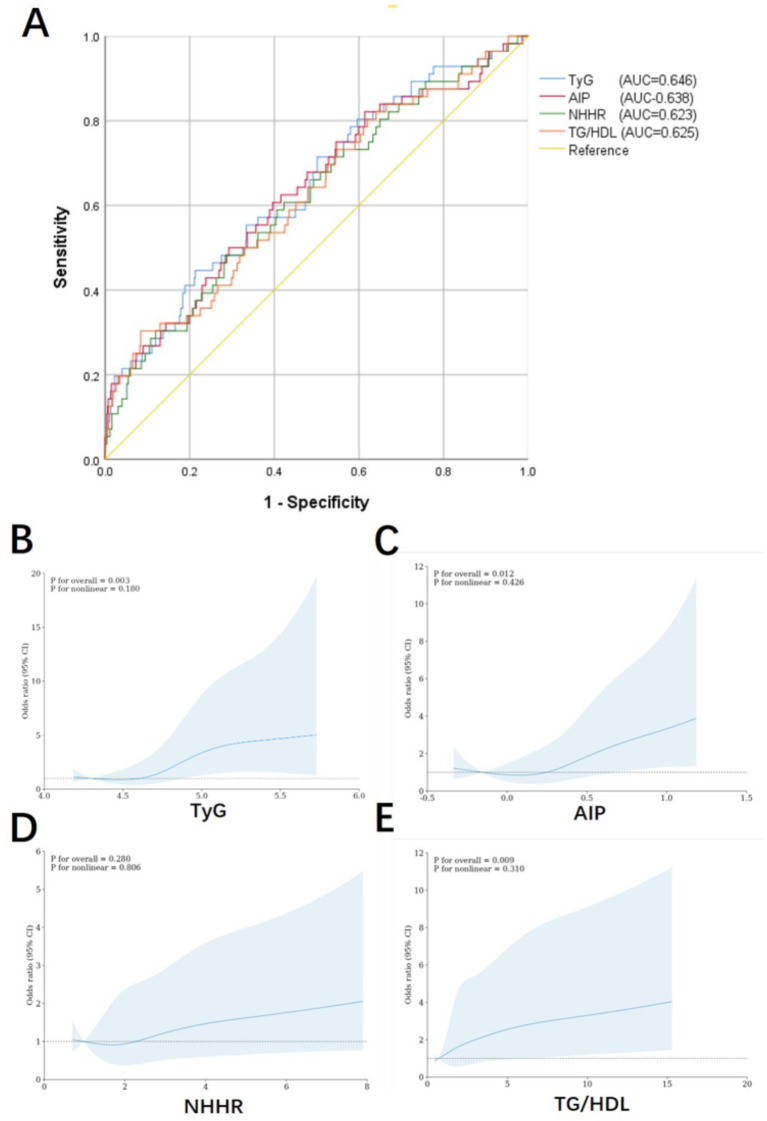
Receiver operating characteristic **(A)** and restricted cubic spline analysis curves **(B-E)** for multi-complications.

Single-factor logistic regression analysis revealed that glycolipid metabolism index including TyG, AIP, NHHR, and TG/HDL-C were closely associated with the occurrence of multiple complications (Table 1) and number of the complications (Table 2). After adjusting for confounding factors (age, sex, mean blood pressure, SpO2, congestive heart failure, race, and diabetes), these indices remained statistically significant (Tables 1, 2). These results underscore the robustness of TyG, AIP, NHHR, and TG/HDL-C as potential predictive factors, demonstrating their independent association with complications following ICH and emphasizing their importance in predicting adverse outcomes in ICH patients. Based on these findings, ROC analysis was conducted to assess the predictive ability of these glycolipid metabolism index for complications post-ICH. When evaluating multi-complications as the outcome, TyG (AUC 0.646), AIP (AUC 0.638), NHHR (AUC 0.626), and TG/HDL-C (AUC 0.625) were assessed (Figure 2A). The results indicated that TyG had relatively better predictive ability for multiple complications. Additionally, RCS analysis was used to identify the relationship between glycolipid metabolism index and multiple complications, showing an increasing trend for TyG, AIP, NHHR, and TG/HDL-C (Figures 2B–E).

**Table 1:** Association between glycolipid metabolism index and multi-complications.

	Univariate logistics analysis			Multivariate logistics analysis		
	OR	95%CI	*P*	OR	95%CI	*P*
TyG	2.794	1.338–5.834	0.006	3.883	1.686–8.941	0.001
AIP	2.897	1.406–5.968	0.004	3.383	1.566–7.309	0.002
NHHR	1.086	0.980–1.203	0.114	1.108	0.996–1.232	0.059
TG/HDL	1.045	1.012–1.080	0.008	1.051	1.015–1.088	0.006

Adjusted for age, sex, mean blood pressure, SpO2, congestive heart failure, race, and diabetes.

OR: Odd ratio.

**Table 2:** Association between glycolipid metabolism index and numbers of complications.

	Univariate linear analysis			Multivariate linear analysis		
	β	95%CI	*P*	β	95%CI	*P*
TyG	0.242	0.087–0.397	0.002	0.264	0.099–0.429	0.002
AIP	0.271	0.121–0.420	<0.001	0.270	0.116–0.424	0.001
NHHR	0.031	0.003–0.058	0.028	0.036	0.008–0.063	0.011
TG/HDL	0.016	0.008–0.023	<0.001	0.016	0.008–0.024	<0.001

Adjusted for age, sex, mean blood pressure, SpO2, congestive heart failure, race, and diabetes.

OR, odd ratio.

Further exploration focused on the relationship between these glycolipid metabolism index and specific complications. Multivariate logistic regression analysis revealed that higher TG/HDL-C was associated with higher risk of deep vein thrombosis (*p* = 0.014), while higher TyG (*p* = 0.002), AIP (*p* = 0.002), and TG/HDL-C (*p* = 0.011) were associated with pulmonary embolism. Higher TyG (*p* = 0.002), AIP (*p* < 0.001), NHHR (*p* = 0.008), and TG/HDL-C (*p* = 0.012) were associated with acute kidney failure (Table 3).

**Table 3:** Association between glycolipid metabolism index and different complication.

	TyG		AIP		NHHR		TH/HDL	
	OR(95%CI)	*P*	OR(95%CI)	*P*	OR(95%CI)	*P*	OR(95%CI)	*P*
Epilepsy (*n* = 46, 6.2%)	1.516(0.583–3.939)	0.393	2.001(0.855–4.685)	0.110	1.061(0.933–1.205)	0.367	1.011(0.979–1.045)	0.503
Alimentary tract complications (*n* = 10, 1.3%)	4.968(0.849–29.085)	0.075	1.735(0.266–11.333)	0.565	0.868(0.501–1.476)	0.601	1.002(0.895–1.122)	0.969
Urinary_tract_infection (*n* = 98, 13.3%)	1.101(0.532–2.276)	0.796	0.885(0.449–1.744)	0.724	0.976(0.842–1.132)	0.751	0.982(0.932–1.034)	0.488
Venous_thrombosis_nec (*n* = 36, 4.9%)	1.395(0.485–4.013)	0.537	1.967(0.776–4.985)	0.154	1.074(0.957–1.205)	0.228	1.037(1.007–1.068)	0.014
Pulmonary_embolism (*n* = 16, 2.1%)	8.246(2.167–31.381)	0.002	6.566(1.997–21.698)	0.002	1.124(0.969–1.303)	0.122	1.041(1.009–1.073)	0.011
Acute_kidney_failure (*n* = 115, 15.6%)	2.867(1.496–5.495)	0.002	3.095(1.698–5.641)	<0.001	1.137(1.034–1.249)	0.008	1.038(1.008–1.069)	0.012
Sepsis (*n* = 13,1.7%)	0.481(0.070–3.283)	0.455	2.816(0.682–11.633)	0.153	1.003(0.726–1.385)	0.988	1.006(0.945–1.070)	0.856

Adjusted for age, sex, mean blood pressure, SpO2, race, and diabetes.

### Relationship between glycolipid metabolism index and mechanical ventilation

As shown in Table 4, single-factor logistic regression analysis revealed significant associations between TyG, AIP, and TG/HDL-C levels and the use of MV. After adjusting for confounding factors, these indices retained their statistical significance. Further analysis explored the relationship between these indices and the duration of MV (Table 5). The results consistently demonstrated that both single-factor and multivariate logistic regression analyses indicated a positive correlation between TyG, AIP, TG/HDL-C levels, and mechanical ventilation duration, establishing them as robust independent predictors.

**Table 4:** Association between glycolipid metabolism index and the use of mechanical ventilation.

	Univariate logistics analysis			Multivariate logistics analysis		
	OR	95%CI	*P*	OR	95%CI	*P*
TyG	3.335	1.966–5.657	<0.001	4.052	2.269–7.237	<0.001
AIP	2.038	1.261–3.293	0.004	2.071	1.242–3.452	0.005
NHHR	1.039	0.949–1.138	0.406	1.045	0.951–1.148	0.363
TG/HDL	1.086	1.018–1.159	0.012	1.088	1.017–1，163	0.014

Adjusted for age, sex, mean blood pressure, SpO2, congestive heart failure, race, and diabetes.

OR: Odd ratio.

**Table 5:** Association between glycolipid metabolism index and the duration of the mechanical ventilation.

	Univariate linear analysis			Multivariate linear analysis		
	*β*	95%CI	*P*	*β*	95%CI	*P*
TyG	52.624	10.275–94.973	0.015	42.803	–3.147–88.152	0.062
AIP	67.807	27.086–108.529	0.001	61.386	18.524–104.248	0.005
NHHR	6.635	1.528–15.743	0.017	7.382	–0.43–14.808	0.051
TG/HDL	2.630	0.755–4.506	0.006	2.591	0.701–4.480	0.007

Adjusted for age, sex, mean blood pressure, SpO2, congestive heart failure, race, and diabetes.

OR: Odd ratio.

### Combined use of ventilators and glycolipid metabolism index to predict multiple complications after ICH

As depicted in Table 6, patients from the original cohort were categorized into two groups based on high and low levels of glycolipid metabolism index, further subdivided into four groups based on the use of MV. Our study revealed that the incidence rates of complications in the High TyG + no Ventilator, Low TyG + Ventilator, and High TyG + Ventilator groups were 3.964-fold, 3.743-fold, and 9.743-fold higher, respectively, compared to the Low TyG + no Ventilator group. For the High AIP + no Ventilator, Low AIP + Ventilator, and High AIP + Ventilator groups, the incidence rates were 8.337-fold, 9.464-fold, and 16.883-fold higher, respectively, compared to the Low AIP + no Ventilator group. Similarly, the High NHHR + no Ventilator, Low NHHR + Ventilator, and High NHHR + Ventilator groups showed incidence rates 2.868-fold, 5.087-fold, and 6.946-fold higher, respectively, compared to the Low NHHR + no Ventilator group. The High TG/HDL + no Ventilator, Low TG/HDL + Ventilator, and High TG/HDL + Ventilator groups exhibited incidence rates 8.095-fold, 9.357-fold, and 16.668-fold higher, respectively, compared to the Low TG/HDL + no Ventilator group. These results indicate that higher levels of glycolipid metabolism index are associated with a greater likelihood of complications. Moreover, this vulnerable population demonstrates a significantly increased risk of complications following MV. This underscores the importance for clinicians to pay closer attention to the occurrence and treatment of complications in such individuals.

**Table 6:** Combined use of ventilators and glycolipid metabolism index to predict multiple complications after ICH.

	Multivariate logistics analysis		
	OR	95%CI	*P*
TyG			
Low TyG + no ventilator	Reference		
High TyG + no Ventilator	3.964	0.743–21.144	0.107
Low TyG + ventilator	3.743	0.823–17.023	0.088
High TyG + Ventilator	9.743	2.273–41.764	0.002
AIP			
Low AIP + no ventilator	Reference		
High AIP + no Ventilator	8.337	0.979–70.974	0.052
Low AIP + ventilator	9.464	1.239–72.287	0.030
High AIP + Ventilator	16.883	2.251–126.623	0.006
NHHR			
Low NHHR + no ventilator	Reference		
High NHHR + no Ventilator	2.868	0.542–25.296	0.216
Low NHHR + ventilator	5.087	1.164–22.237	0.031
High NHHR + Ventilator	6.946	1.603–30.106	0.010
TG/HDL			
Low TG/HDL + no ventilator	Reference		
High TG/HDL + no Ventilator	8.095	0.951–68.900	0.056
Low TG/HDL + ventilator	9.357	1.225–71.499	0.031
High TG/HDL + Ventilator	16.668	2.222–125.030	0.006

Adjusted for age, sex, mean blood pressure, SpO2, congestive heart failure, race, and diabetes.

OR, odd ratio.

## Discussion

In this retrospective cohort study including 733 ICU-admitted ICU patients, 7.64% patients experienced at least two medical complications. The incidence rates were as follows: seizures 6.28%, gastrointestinal ulcers and bleeding 1.36%, urinary tract infections 13.37%, deep vein thrombosis 4.91%, pulmonary embolism 2.18%, acute kidney injury 15.69%, and sepsis 1.77%. Additionally, MV use increased the risk of multiple complications. These findings align with previous research trends (5). Our study revealed associations between glycolipid metabolism index and multiple ICH-related complications. TyG, AIP, and TG/HDL-C were identified as independent predictors of pulmonary embolism, while all four glycolipid metabolism indices independently predicted acute kidney injury. Furthermore, we demonstrated significant correlations between glycolipid metabolism index levels and duration of MV in the ICU. Therefore, these results suggest that glycolipid metabolism index, as readily available index, could serve as reliable predictors for complications and MV duration among ICH patients in the ICU.

Diabetes and obesity, as systemic metabolic disorders, have been extensively studied and linked to infectious diseases, thrombosis, and multiple organ dysfunction. Previous research has established significant correlations between TyG and in-hospital mortality, ICU mortality, and overall mortality rates in critically ill patients with ICH (16, 17). This underscores the value of glycolipid metabolism index in predicting outcomes for ICH patients. However, there is a lack of studies investigating the role of TyG and other glycolipid metabolism index in predicting post-ICH complications. Our study is the first to demonstrate a close association between these glycolipid metabolism index and the development of multiple complications following ICH.

Deep vein thrombosis and pulmonary embolism are common thrombotic events following ICH, closely associated with poor prognosis (18). Certain coagulation index such as D-dimer and fibrinogen (19, 20), as well as inflammatory index including platelet-to-lymphocyte ratio and serum C-reactive protein (19, 21), have been validated as predictors of deep vein thrombosis post-ICH. Given the close relationship between dyslipidemia and thrombosis, various glycolipid metabolism index such as TyG, AIP, NHHR, and TG/HDL-C may serve as potential predictors of thrombosis. Our study demonstrates TG/HDL-C as a predictor of deep vein thrombosis, whereas TyG, AIP, and TG/HDL-C predict pulmonary embolism occurrence, likely due to dyslipidemia-induced endothelial dysfunction promoting coagulation factor activation and thrombus formation (22). Additionally, dyslipidemia correlates closely with inflammatory response, potentially increasing thrombotic risk indirectly through inflammation (23). Acute kidney injury is also a common complication following ICH, complicating the hospital course and associated with increased mortality and adverse discharge events (24). Early detection of acute kidney injury is crucial in this population. Previous research has identified the TyG as a robust and independent predictor of acute kidney injury incidence and renal outcomes in critically ill heart failure and sepsis patients (25), consistent with our findings. Furthermore, we assessed the predictive ability of other glycolipid metabolism index including AIP, NHHR, and TG/HDL-C, all of which significantly correlated with the incidence of acute kidney injury post-ICH.

Diabetes mellitus patients face an increased risk of hospital-acquired infections 2 to 4 times higher compared to the general population (14, 26, 27). Similarly, obesity also elevates the risk of hospital infections, including postoperative and other nosocomial infections (28, 29). This may be attributed to the metabolic disturbances such as hyperglycemia and dyslipidemia affecting immune cell function (30, 31). Moreover, metabolic dysregulation can impair vascular function, increase intestinal permeability, and disrupt gut microbiota, all contributing to the occurrence and spread of infections (14, 32). Currently, effective predictors of hospital-acquired infections following ICH remain scarce. Limited studies have shown predictive capabilities of certain markers. A retrospective study confirmed that lymphocytopenia on admission is associated with nosocomial urinary tract infections and significantly shortens the time to infection occurrence (33). Furthermore, inflammatory index (systemic inflammatory response index) and nutritional index (controlling nutritional status and prognostic nutritional index) independently correlate with stroke-associated pneumonia (34). While some studies have demonstrated the close association of glycolipid metabolism markers with mortality in infectious diseases such as coronavirus disease 2019 (35) and sepsis (36), there is currently no research assessing the predictive ability of these markers for infectious complications following ICH. Unfortunately, our study indicates that glycolipid metabolism index (TyG, AIP, NHHR, and TG/HDL-C) do not effectively predict the occurrence of urinary tract infections, sepsis, or gastrointestinal ulceration and bleeding post-ICH. However, this may be influenced by the low incidence of such complications in our cohort. Considering the pathological mechanisms linking metabolic disturbances to infections, the predictive value of these glycolipid metabolism-related markers in such complications cannot be ruled out and warrants further investigation.

Severe cerebral hemorrhage can lead to cerebral edema affecting the respiratory center, resulting in central respiratory failure, hypoxemia, and respiratory alkalosis. Additionally, concurrent pulmonary infections and airway mucus obstruction often hinder effective correction of hypoxemia with conventional oxygen therapy (37, 38). Therefore, MV is crucial for stabilizing respiratory and circulatory function following ICH. However, research indicates an increased risk of medical complications within 24 h of MV use in these patients (6). Thus, meticulous attention should be given to the occurrence of complications in ICH patients requiring MV. Our study suggests that in ICU populations with cerebral hemorrhage, glycolipid metabolism index such as TyG, AIP, and TG/HDL-C are associated with the use and duration of MV. We hypothesize that this association may be linked to obesity prevalent in patients with elevated glycolipid metabolism index, who may require endotracheal intubation for effective correction of post-ICH hypoxia due to their unique anatomical and physiological characteristics. Moreover, systemic inflammatory responses and pulmonary dysfunction resulting from metabolic dysregulation could contribute to difficulties in weaning from MV (39). Importantly, our findings further indicate that elevated glycolipid metabolism index (including TyG, AIP, NHHR, and TG/HDL-C) in ICH patients increase the probability of developing more complications with MV. Hence, enhanced vigilance and management of complications are crucial for this patient population.

This study has several limitations. Firstly, it is a retrospective study, which is susceptible to selection bias. However, we strictly defined the inclusion criteria to accurately reflect real-world scenarios as much as possible. Secondly, our data were sourced from the MIMIC-IV database, relying on the accuracy of medical records, and the completeness of data varied to some extent. Some complications occurred infrequently, which resulted in lower statistical power. Additionally, due to the limitations of the sample size in public databases and the difficulty in obtaining information on related confounding factors (such as hematoma volume and progression), this study did not determine the clinical threshold values of these indices. Further investigation through larger-scale prospective studies is needed. Furthermore, our study only explored the predictive value of glycolipid metabolism indices at a single time point for the prognosis of ICH patients. However, the dynamic changes in these indices will be an important direction for future research. It is worth noting that future studies should not only focus on the traditional TyG, but also consider the growing attention given to several TyG-derived indices in recent years, such as the TyG waist circumference index, TyG body mass index, and TyG waist-to-height ratio index (40). These derivatives combine TyG with other physical parameters, providing more comprehensive metabolic information and potentially enhancing predictive power. Unfortunately, due to missing data in the MIMIC-IV database, we were unable to explore the benefits of these indices in this study. Therefore, future research should further validate the effectiveness of these derivatives in different clinical populations and explore their underlying mechanisms, particularly how optimizing metabolic status can reduce the occurrence of complications.

## Conclusion

In summary, our study findings indicate that glycolipid metabolism markers, particularly TyG, AIP, and TG/HDL-C, can predict both the use of mechanical ventilation in ICH patients and serve as independent predictors of complications in this population.

## Data Availability

Publicly available datasets were analyzed in this study. This data can be found at: MIMIC-IV database (version 2.2), https://physionet.org/content/mimiciv/2.2/.
