# Supplementary material for: Prognostic value of glycolipid metabolism index on complications and mechanical ventilation in intensive care unit patients with intracerebral hemorrhage: a retrospective cohort study using the MIMIC-IV database
Source: Front Neurol. 2025 Feb 25;16:1516627. doi: 10.3389/fneur.2025.1516627 (PMC11893385; doi:10.3389/fneur.2025.1516627)
Supplement: Supplementary file 1 [file Table_1.docx]

**Summary descriptives table by groups of `ventilator'**

|  | **[ALL]** | **no** | **yes** | **p.overall** |
| --- | --- | --- | --- | --- |
|  | ***N=733*** | ***N=238*** | ***N=495*** |  |
| **Gender:** |  |  |  | 0.519 |
| male | 396 (54.02%) | 124 (52.10%) | 272 (54.95%) |  |
| female | 337 (45.98%) | 114 (47.90%) | 223 (45.05%) |  |
| **Weight** | 80.68 (23.29) | 77.02 (25.77) | 81.53 (22.63) | 0.160 |
| **Age** | 70.36 (14.88) | 70.12 (15.70) | 70.47 (14.48) | 0.776 |
| **Race （WHITE=1，BLACK=2，ASIAN=3，HISPANIC=4, other=5, UNKNOWN=6）:** |  |  |  | . |
| 1 | 441 (60.16%) | 148 (62.18%) | 293 (59.19%) |  |
| 2 | 72 (9.82%) | 30 (12.61%) | 42 (8.48%) |  |
| 3 | 23 (3.14%) | 11 (4.62%) | 12 (2.42%) |  |
| 4 | 21 (2.86%) | 10 (4.20%) | 11 (2.22%) |  |
| 5 | 42 (5.73%) | 11 (4.62%) | 31 (6.26%) |  |
| 6 | 134 (18.28%) | 28 (11.76%) | 106 (21.41%) |  |
| **The length of stay in the intensive care unit** | 6.55 (6.91) | 3.67 (2.70) | 7.94 (7.84) | **<0.001** |
| **GCS** | 14.09 (1.69) | 14.09 (1.44) | 14.08 (1.79) | 0.938 |
| **Respiratory rate** | 18.69 (5.07) | 18.36 (5.45) | 18.85 (4.87) | 0.236 |
| **Heart rate** | 81.55 (17.00) | 78.72 (15.32) | 82.91 (17.61) | **0.001** |
| **SBP** | 139.54 (23.58) | 137.50 (20.09) | 140.53 (25.05) | 0.080 |
| **DBP** | 76.61 (17.85) | 77.60 (15.19) | 76.14 (18.99) | 0.263 |
| **MBP** | 93.35 (17.89) | 94.55 (18.05) | 92.77 (17.80) | 0.210 |
| **Spo2** | 97.37 (3.53) | 97.61 (2.36) | 97.26 (3.97) | 0.134 |
| **WBC** | 10.59 (3.91) | 9.44 (3.71) | 11.15 (3.89) | **<0.001** |
| **Platelet** | 216.89 (76.98) | 220.76 (75.96) | 215.03 (77.47) | 0.343 |
| **RBC** | 4.13 (0.65) | 4.19 (0.61) | 4.10 (0.66) | 0.064 |
| **Hemoglobin** | 12.34 (1.89) | 12.40 (1.76) | 12.31 (1.95) | 0.534 |
| **Potassium** | 4.00 (0.62) | 4.04 (0.52) | 3.98 (0.66) | 0.184 |
| **Sodium** | 139.65 (4.06) | 139.68 (3.66) | 139.64 (4.24) | 0.884 |
| **Calcium** | 8.74 (0.74) | 8.91 (0.59) | 8.67 (0.80) | **<0.001** |
| **Bun** | 19.08 (13.23) | 17.63 (9.95) | 19.78 (14.50) | **0.020** |
| **Creatinine** | 1.08 (0.85) | 1.00 (0.61) | 1.12 (0.94) | **0.029** |
| **Glucose** | 140.23 (62.79) | 126.84 (49.45) | 146.67 (67.38) | **<0.001** |
| **HDL-C** | 51.19 (18.34) | 53.29 (17.01) | 50.19 (18.88) | **0.026** |
| **Total cholesterol** | 165.27 (45.25) | 170.98 (43.56) | 162.52 (45.83) | **0.016** |
| **Triglycerides** | 125.55 (135.42) | 109.70 (73.06) | 133.13 (156.23) | **0.006** |
| **TyG** | 8.82 (0.66) | 8.66 (0.57) | 8.90 (0.68) | **<0.001** |
| **AIP** | 0.34 (0.34) | 0.28 (0.30) | 0.36 (0.35) | **0.002** |
| **NHHR** | 2.60 (1.88) | 2.52 (1.50) | 2.64 (2.04) | 0.353 |
| **TG/HDL-C** | 3.28 (6.49) | 2.51 (2.26) | 3.64 (7.71) | **0.003** |
| **INR** | 1.21 (0.31) | 1.19 (0.35) | 1.21 (0.28) | 0.344 |
| **PT** | 13.22 (3.32) | 12.99 (3.79) | 13.33 (3.07) | 0.239 |
| **PTT** | 29.41 (10.77) | 28.75 (7.14) | 29.73 (12.11) | 0.187 |
| **Charlson comorbidity index** | 5.80 (2.51) | 5.58 (2.56) | 5.90 (2.49) | 0.111 |
| **Age score** | 2.53 (1.33) | 2.51 (1.36) | 2.54 (1.33) | 0.801 |
| **Diabetes:** |  |  |  | 0.852 |
| no | 528 (72.03%) | 173 (72.69%) | 355 (71.72%) |  |
| yes | 205 (27.97%) | 65 (27.31%) | 140 (28.28%) |  |
| **Renal disease:** |  |  |  | 0.813 |
| no | 633 (86.36%) | 204 (85.71%) | 429 (86.67%) |  |
| yes | 100 (13.64%) | 34 (14.29%) | 66 (13.33%) |  |
| **Myocardia infarct:** |  |  |  | 0.108 |
| no | 650 (88.68%) | 218 (91.60%) | 432 (87.27%) |  |
| yes | 83 (11.32%) | 20 (8.40%) | 63 (12.73%) |  |
| **Peripheral vascular disease:** |  |  |  | **0.019** |
| no | 680 (92.77%) | 229 (96.22%) | 451 (91.11%) |  |
| yes | 53 (7.23%) | 9 (3.78%) | 44 (8.89%) |  |
| **Dementia** | 0.06 (0.24) | 0.09 (0.29) | 0.04 (0.21) | **0.023** |
| **Chronic pulmonary disease:** |  |  |  | **0.001** |
| no | 634 (86.49%) | 221 (92.86%) | 413 (83.43%) |  |
| yes | 99 (13.51%) | 17 (7.14%) | 82 (16.57%) |  |
| **Mild liver disease:** | 0.03 (0.17) | 0.03 (0.17) | 0.03 (0.18) | 0.830 |
| **Epilepsy:** |  |  |  | **0.036** |
| no | 687 (93.72%) | 230 (96.64%) | 457 (92.32%) |  |
| yes | 46 (6.28%) | 8 (3.36%) | 38 (7.68%) |  |
| **Gastrointestinal ulcer and hemorrhage:** |  |  |  | 1.000 |
| no | 723 (98.64%) | 235 (98.74%) | 488 (98.59%) |  |
| yes | 10 (1.36%) | 3 (1.26%) | 7 (1.41%) |  |
| **Urinary tract infection:** |  |  |  | 0.317 |
| no | 635 (86.63%) | 211 (88.66%) | 424 (85.66%) |  |
| yes | 98 (13.37%) | 27 (11.34%) | 71 (14.34%) |  |
| **Deep vein thrombosis:** |  |  |  | 0.244 |
| no | 697 (95.09%) | 230 (96.64%) | 467 (94.34%) |  |
| yes | 36 (4.91%) | 8 (3.36%) | 28 (5.66%) |  |
| **Pulmonary embolism:** |  |  |  | **0.046** |
| no | 717 (97.82%) | 237 (99.58%) | 480 (96.97%) |  |
| yes | 16 (2.18%) | 1 (0.42%) | 15 (3.03%) |  |
| **Acute kidney failure:** |  |  |  | **<0.001** |
| no | 618 (84.31%) | 219 (92.02%) | 399 (80.61%) |  |
| yes | 115 (15.69%) | 19 (7.98%) | 96 (19.39%) |  |
| **Sepsis:** |  |  |  | 0.071 |
| no | 720 (98.23%) | 237 (99.58%) | 483 (97.58%) |  |
| yes | 13 (1.77%) | 1 (0.42%) | 12 (2.42%) |  |
| **Complication number**: |  |  |  | **0.002** |
| ≤1 | 677 (92.36%) | 231 (97.06%) | 446 (90.10%) |  |
| ≥2 | 56 (7.64%) | 7 (2.94%) | 49 (9.90%) |  |
