# Supplementary material for: Prognostic value of glycolipid metabolism index on complications and mechanical ventilation in intensive care unit patients with intracerebral hemorrhage: a retrospective cohort study using the MIMIC-IV database
Source: Front Neurol. 2025 Feb 25;16:1516627. doi: 10.3389/fneur.2025.1516627 (PMC11893385; doi:10.3389/fneur.2025.1516627)
Supplement: Supplementary file 2 [file Table_2.docx]

Summary descriptives table by groups of `Complications'

|  | **[ALL]** | **no** | **yes** | **p.overall** |
| --- | --- | --- | --- | --- |
|  | ***N=733*** | ***N=677*** | ***N=56*** |  |
| **Gender:** |  |  |  | 1.000 |
| male | 396 (54.02%) | 366 (54.06%) | 30 (53.57%) |  |
| female | 337 (45.98%) | 311 (45.94%) | 26 (46.43%) |  |
| **Weight** | 80.68 (23.29) | 79.75 (22.68) | 88.26 (26.96) | 0.050 |
| **Age** | 70.36 (14.88) | 70.29 (14.85) | 71.13 (15.39) | 0.697 |
| **Race （WHITE=1，BLACK=2，ASIAN=3，HISPANIC=4, other=5, UNKNOWN=6）:** |  |  |  | 0.361 |
| 1 | 441 (60.16%) | 411 (60.71%) | 30 (53.57%) |  |
| 2 | 72 (9.82%) | 64 (9.45%) | 8 (14.29%) |  |
| 3 | 23 (3.14%) | 22 (3.25%) | 1 (1.79%) |  |
| 4 | 21 (2.86%) | 21 (3.10%) | 0 (0.00%) |  |
| 5 | 42 (5.73%) | 40 (5.91%) | 2 (3.57%) |  |
| 6 | 134 (18.28%) | 119 (17.58%) | 15 (26.79%) |  |
| **The length of stay in the intensive care unit** | 6.55 (6.91) | 5.91 (5.89) | 14.27 (12.01) | **<0.001** |
| **GCS** | 14.09 (1.69) | 14.09 (1.65) | 14.04 (2.05) | 0.848 |
| **Respiratory rate** | 18.69 (5.07) | 18.61 (5.05) | 19.66 (5.25) | 0.154 |
| **Heart rate** | 81.55 (17.00) | 81.29 (16.51) | 84.73 (22.06) | 0.257 |
| **SBP** | 139.54 (23.58) | 139.91 (23.76) | 135.18 (20.98) | 0.114 |
| **DBP** | 76.61 (17.85) | 76.69 (17.92) | 75.71 (17.12) | 0.685 |
| **MBP** | 93.35 (17.89) | 93.44 (18.05) | 92.29 (15.91) | 0.608 |
| **Spo2** | 97.37 (3.53) | 97.48 (2.74) | 96.02 (8.47) | 0.203 |
| **WBC** | 10.59 (3.91) | 10.49 (3.77) | 11.78 (5.21) | 0.075 |
| **Platelet** | 216.89 (76.98) | 218.17 (77.24) | 201.39 (72.58) | 0.103 |
| **RBC** | 4.13 (0.65) | 4.14 (0.63) | 3.99 (0.77) | 0.159 |
| **Hemoglobin** | 12.34 (1.89) | 12.38 (1.87) | 11.86 (2.13) | 0.076 |
| **Potassium** | 4.00 (0.62) | 3.99 (0.58) | 4.16 (0.94) | 0.181 |
| **Sodium** | 139.65 (4.06) | 139.60 (3.86) | 140.30 (5.93) | 0.384 |
| **Calcium** | 8.74 (0.74) | 8.75 (0.73) | 8.63 (0.87) | 0.291 |
| **Bun** | 19.08 (13.23) | 18.51 (11.77) | 25.96 (23.93) | **0.025** |
| **Creatinine** | 1.08 (0.85) | 1.06 (0.85) | 1.30 (0.82) | **0.041** |
| **Glucose** | 140.23 (62.79) | 140.46 (64.07) | 137.39 (44.77) | 0.636 |
| **HDL-C** | 51.19 (18.34) | 51.34 (18.13) | 49.43 (20.77) | 0.507 |
| **Total cholesterol** | 165.27 (45.25) | 164.81 (43.43) | 170.82 (63.54) | 0.490 |
| **Triglycerides** | 125.55 (135.42) | 119.31 (91.53) | 200.79 (366.99) | 0.103 |
| **TyG** | 8.82 (0.66) | 8.80 (0.64) | 9.06 (0.78) | **0.021** |
| **AIP** | 0.34 (0.34) | 0.33 (0.33) | 0.46 (0.44) | **0.024** |
| **NHHR** | 2.60 (1.88) | 2.57 (1.76) | 3.00 (3.01) | 0.290 |
| **TG/HDL-C** | 3.28 (6.49) | 2.97 (3.74) | 6.98 (19.30) | 0.126 |
| **INR** | 1.21 (0.31) | 1.20 (0.30) | 1.30 (0.33) | **0.041** |
| **PT** | 13.22 (3.32) | 13.15 (3.28) | 14.07 (3.64) | 0.077 |
| **PTT** | 29.41 (10.77) | 29.13 (9.47) | 32.62 (20.39) | 0.218 |
| **Ventilator:** |  |  |  | **0.002** |
| no | 238 (32.47%) | 231 (34.12%) | 7 (12.50%) |  |
| yes | 495 (67.53%) | 446 (65.88%) | 49 (87.50%) |  |
| **Ventilation hours** | 109.81 (164.68) | 93.98 (142.50) | 253.96 (259.44) | **<0.001** |
| **Charlson comorbidity index** | 5.80 (2.51) | 5.75 (2.48) | 6.29 (2.89) | 0.187 |
| **Age score** | 2.53 (1.33) | 2.52 (1.33) | 2.70 (1.44) | 0.370 |
| **Diabetes:** |  |  |  | 0.719 |
| no | 528 (72.03%) | 486 (71.79%) | 42 (75.00%) |  |
| yes | 205 (27.97%) | 191 (28.21%) | 14 (25.00%) |  |
| **Renal disease:** |  |  |  | **0.001** |
| no | 633 (86.36%) | 593 (87.59%) | 40 (71.43%) |  |
| yes | 100 (13.64%) | 84 (12.41%) | 16 (28.57%) |  |
| **Myocardial infarct:** |  |  |  | 0.343 |
| no | 650 (88.68%) | 603 (89.07%) | 47 (83.93%) |  |
| yes | 83 (11.32%) | 74 (10.93%) | 9 (16.07%) |  |
| **Peripheral vascular disease:** |  |  |  | 0.589 |
| no | 680 (92.77%) | 629 (92.91%) | 51 (91.07%) |  |
| yes | 53 (7.23%) | 48 (7.09%) | 5 (8.93%) |  |
| **Dementia** | 0.06 (0.24) | 0.06 (0.24) | 0.04 (0.19) | 0.327 |
| **Chronic pulmonary disease:** |  |  |  | 0.979 |
| no | 634 (86.49%) | 585 (86.41%) | 49 (87.50%) |  |
| yes | 99 (13.51%) | 92 (13.59%) | 7 (12.50%) |  |
| **Mild liver disease** | 0.03 (0.17) | 0.03 (0.17) | 0.05 (0.23) | 0.442 |
| **Epilepsy:** |  |  |  | **<0.001** |
| no | 687 (93.72%) | 647 (95.57%) | 40 (71.43%) |  |
| yes | 46 (6.28%) | 30 (4.43%) | 16 (28.57%) |  |
| **Gastrointestinal ulcer and hemorrhage** |  |  |  | **<0.001** |
| no | 723 (98.64%) | 673 (99.41%) | 50 (89.29%) |  |
| yes | 10 (1.36%) | 4 (0.59%) | 6 (10.71%) |  |
| **Urinary tract infection:** |  |  |  | **<0.001** |
| no | 635 (86.63%) | 609 (89.96%) | 26 (46.43%) |  |
| yes | 98 (13.37%) | 68 (10.04%) | 30 (53.57%) |  |
| **Deep vein thrombosis:** |  |  |  | **<0.001** |
| no | 697 (95.09%) | 661 (97.64%) | 36 (64.29%) |  |
| yes | 36 (4.91%) | 16 (2.36%) | 20 (35.71%) |  |
| **Pulmonary embolism:** |  |  |  | **<0.001** |
| no | 717 (97.82%) | 675 (99.70%) | 42 (75.00%) |  |
| yes | 16 (2.18%) | 2 (0.30%) | 14 (25.00%) |  |
| **Acute kidney failure:** |  |  |  | **<0.001** |
| no | 618 (84.31%) | 596 (88.04%) | 22 (39.29%) |  |
| yes | 115 (15.69%) | 81 (11.96%) | 34 (60.71%) |  |
| **Sepsis:** |  |  |  | **<0.001** |
| no | 720 (98.23%) | 673 (99.41%) | 47 (83.93%) |  |
| yes | 13 (1.77%) | 4 (0.59%) | 9 (16.07%) |  |
